# Advances in melt electrowriting for cardiovascular applications

**DOI:** 10.3389/fbioe.2024.1425073

**Published:** 2024-09-17

**Authors:** Kilian Maria Arthur Mueller, Salma Mansi, Elena M. De-Juan-Pardo, Petra Mela

**Affiliations:** ^1^ Technical University of Munich, TUM School of Engineering and Design, Department of Mechanical Engineering, Chair of Medical Materials and Implants, Munich Institute of Biomedical Engineering (MIBE), Munich Institute of Integrated Materials, Energy and Process Engineering (MEP), Munich, Germany; ^2^ T3mPLATE, Harry Perkins Institute of Medical Research, Queen Elizabeth II Medical Centre and University of Western Australia Centre for Medical Research, The University of Western Australia, Perth, WA, Australia; ^3^ School of Engineering, The University of Western Australia, Perth, WA, Australia; ^4^ Curtin Medical School, Curtin University, Perth, WA, Australia

**Keywords:** melt electrowriting, tissue engineering, heart valve, cardiac patch, vascular graft, capillary network, cardiovascular

## Abstract

Melt electrowriting (MEW) is an electric-field-assisted additive biofabrication technique that has brought significant advancements to bioinspired scaffold design for soft tissue engineering and beyond. Owing to its targeted microfiber placement, MEW has become a powerful platform technology for the fabrication of *in vitro* disease models up to functional biohybrid constructs that are investigated *in vivo* to reach clinical translation soon. This work provides a concise overview of this rapidly evolving field by highlighting the key contributions of MEW to cardiovascular tissue engineering. Specifically, we i) pinpoint the methods to introduce microvascular networks in thick 3D constructs benefitting from (sacrificial) MEW microfibers, ii) report MEW-based concepts for small-diameter vascular grafts and stents, iii) showcase how contracting cardiac tissues can profit from the tunable structure–property relationship of MEW scaffolds, and iv) address how complete regenerative heart valves can be built on complex fiber scaffold architectures that recapitulate J-shaped tensile properties and tissue heterogeneity. Lastly, we touch on novel biomaterial advancements and discuss the technological challenges of MEW to unlock the full potential of this transformative technology.

## 1 Introduction

Melt electrowriting (MEW) is a powerful fiber-forming biofabrication strategy that combines electrically driven fiber jet formation with digitally controlled jet deposition to form highly ordered microfibrous architectures following the layer-by-layer paradigm of additive manufacturing.

**FIGURE 1:**
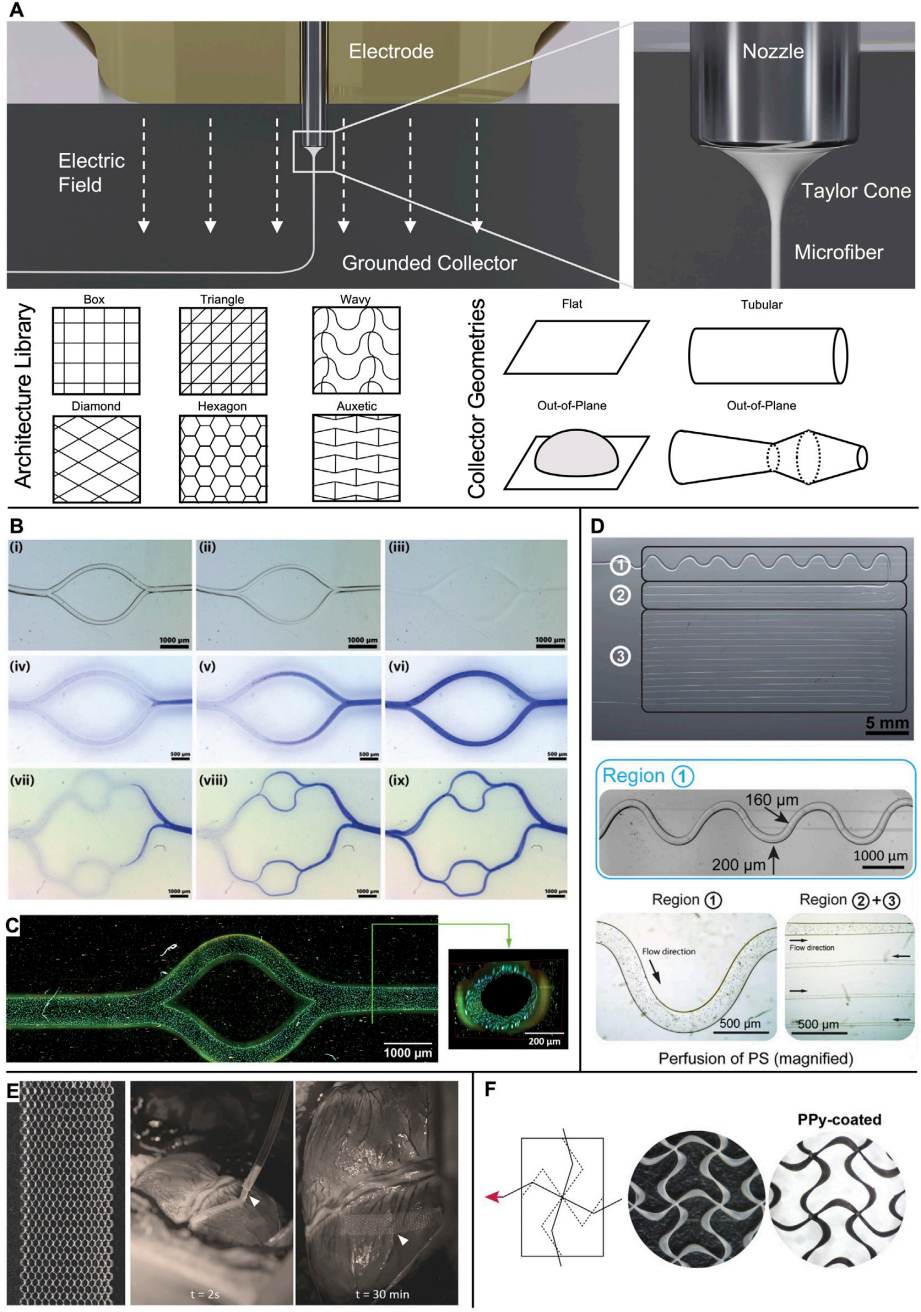
Microchannels and cardiac patches obtained via MEW. **(A)** In MEW, a polymer melt is extruded via a charged nozzle and transforms into a fiber jet that undergoes significant thinning in diameter while traveling towards the collector, where it is deposited in direct writing mode according to computer-coded paths. A wide range of fiber pattern architectures has been established and realized onto various collector geometries to result in flat, tubular, or out-of-plane scaffolds. **(B)** Examples of bifurcating channel networks. i-iii) time-lapse of the dissolution of P*cyclo*PrOx fibers and iv-ix) progressing perfusion of the branching channels. **(C)** Multiphoton image of an endothelialized microchannel. Cytoskeleton: Alexa Fluor 488 Phalloidin; nuclei: Hoechst 33342. **(D)** Microchannels from dissolved isomalt in PDMS. The channel diameter was controlled via the fiber diameter from 200 µm (region 1) to 30 µm (region 3). Perfusion was demonstrated via polystyrene (PS) microparticles. **(E)** Detailed image of the as-fabricated hexagonal scaffold and *in vivo* placement of the patch onto a porcine heart. **(F)** Auxetic scaffold design before (white PCL fibers) and after coating with electroconductive PPy (black appearance). **(B, C)** Adapted with permission from Ryma et al. (2022), CC BY-NC 4.0. **(D)** Adapted with permission from Nadernezhad et al. (2021), CC BY-NC 4.0. **(E)** Adapted with permission from Castilho et al. (2018), Wiley. **(F)** Adapted with permission from Olvera et al. (2020), Wiley.

In MEW, a polymer melt is extruded pneumatically from a syringe through a metal nozzle (Eichholz et al., 2022; Lanaro et al., 2021; Mieszczanek et al., 2021b) (Figure 1A) or, as recently proposed, via a filament-based system (Luposchainsky et al., 2022; Mueller et al., 2023a; Reizabal et al., 2023). An electric potential applied to the nozzle transforms the extruded melt into a Taylor cone from which a fiber jet emerges that travels to the oppositely charged collector. The electric field stabilizes the fiber during the flight phase and leads to a significant reduction of its diameter. Collecting the fiber jet onto a flat or tubular target that moves with a speed matching the flight speed of the jet enables fiber deposition in a direct-writing mode along computer-controlled paths so that a three-dimensional structure is formed by repeatedly stacking fiber layers (Robinson et al., 2019). To date, a wide range of fiber architectures have been melt-electrowritten onto flat and tubular collector geometries (Robinson et al., 2019), and some examples of out-of-plane designs have been reported (Brooks-Richards et al., 2022; Luposchainsky et al., 2022; Mueller et al., 2023a; O’Connell et al., 2021; Peiffer et al., 2020; Saha et al., 2021; Saidy et al., 2020) (Figure 1A).

Since its conceptualization (Brown et al., 2011; Dalton et al., 2008; 2007), MEW has been employed to produce scaffolds in a vast array of studies on engineering *inter alia* bone, neural, and cartilage tissue (Robinson et al., 2019). This review specifically addresses MEW as a versatile technology platform to solve challenges in tissue engineering (TE) and regenerative medicine for the cardiovascular system, including capillary networks, vascular grafts, cardiac patches, and heart valves.

## 2 Capillary networks

Cell-laden constructs with a thickness larger than the diffusion limit of oxygen suffer from inadequate nutrient supply and waste removal and hence require microvascularization to avoid necrosis. To this end, various approaches to templating microchannels within hydrogels have been explored based on precisely deposited sacrificial MEW fibers.

Ryma et al. (2022) reported a print-and-fuse strategy via MEW of poly(2-cyclopropyl-2-oxazoline) (P*cyclo*PrOx) to form microchannels in various hydrogels, including gelatin-methacrylol (GelMA), agarose, alginate, and gelatin. P*cyclo*PrOx fibers (87–275 µm diameter) were deposited as multilayered network template, fused via water-induced plasticity, and subsequently dissolved to result in bifurcating channels that follow Murray’s law. A functional endothelial monolayer was obtained within 3 days by static seeding followed by fluid perfusion (Figures 1B,C).

Wang et al. (2020) embedded PCL MEW microfibers (50–220 µm diameter) in poly(ethylene glycol) (PEG) hydrogels as sacrificial channel templates. Dissolving the fibers with 90% acetone resulted in the creation of microchannels, which were then decorated with cell adhesion peptides to guide cell growth into the channels. Physical removal of PCL microfibers embedded in GelMA has also been shown, with the added benefit of obtaining a perfusable channel network within a fiber-reinforced hydrogel when fibers were selectively removed (Liu et al., 2023).

Nadernezhad et al. (2021) pioneered MEW of the water-soluble sugar isomalt as a fugitive channel template (30–200 μm diameter) in polydimethylsiloxane (PDMS) molds for microfluidic model systems (Figure 1D).

A different approach to induce vascularization was undertaken by Bertlein et al. (2018), who leveraged the preferential adhesion of human umbilical vein endothelial cells (HUVECs) along PCL fibers to provide initial orientation for the formation of capillary-like structures in a fibronectin and gelatin matrix with HUVECs and human dermal fibroblasts. When the pore size of the fibrous scaffold exceeded 350 µm, additional neovascular-like structures formed within 7 days to counteract the hypoxic conditions in the pore center.

## 3 Cardiac patches

Cardiac patches are a promising strategy to restore myocardial contractility of infarcted tissue. A clinically successful patch must fulfill very stringent requirements: support the large multiaxial strains of the myocardium, comply with its electroconductivity, and provide the potential to deliver cells along with growth factors to the host tissue. In this context, multiple MEW fiber architectures have been investigated to profit from both cell guidance via geometrical cues and a tunable mechanical structure-property relationship.

Zhang et al. (2023) manufactured box-pore scaffolds (fiber spacing 60–100 µm) from polylactic acid (PLA) and seeded them with human induced pluripotent stem cell-derived cardiomyocytes (iPSC-CM). The MEW scaffold guided the sarcomere formation along its fibers, leading to ordered engineered tissues with synchronous calcium transients.

Castilho et al. (2018) exploited the potential of hexagonal pore scaffold designs for large elastic deformations. These hexagonal scaffolds were able to undergo 35%–40% strain before plastic deformation and to store elastic strain energy ≈20–40 times higher than box-pore scaffolds. However, the hexagonal scaffolds were still stiffer than native cardiac tissue (10–20 kPa at early diastole and 200–500 kPa at late diastole) (Nakano et al., 1990). After 7 days of culture of iPSC-CM encapsulated in a scaffold consisting of collagen-based hydrogel combined with a MEW mesh with hexagonal pores, synchronous contraction was observed along with enhanced cell alignment and sarcomere content, as well as an increase in cardiac maturation-related markers when compared to boxed scaffolds. The patches successfully recovered their shape after epicardial delivery on a beating porcine heart via a catheter-like 1.5 mm diameter tube owing to their superior elastic compliance (Figure 1E). In a follow-up study, the MEW mesh was filled with two different hydrogels via extrusion-based bioprinting: first, a myocardial bioink containing iPSC-CM + human fetal cardiac fibroblasts (hfCFs) and second, a vascular bioink with HUVECs + hfCFs that was patterned according to the shape of the left anterior descending artery to serve as pre-vascular pathway (Ainsworth et al., 2023). However, when straining such hexagonal scaffolds in the axial direction, they suffer from contraction in the transversal direction (Castilho et al., 2018). Auxetic scaffold designs, such as a missing-rib model, are characterized by a negative Poisson’s ratio and, therefore, allow large biaxial strains as needed for cardiac patches (Figure 1F) (Olvera et al., 2020). Furthermore, by tuning the geometrical features of the repeating cell unit, an anisotropy ratio of effective stiffness close to that reported for human myocardium (1.9–3.9) was obtained while still presenting sufficient elasticity (Engelmayr et al., 2008; Montgomery et al., 2017; Neal et al., 2013; Park et al., 2011). In the second step, these PCL scaffolds were coated with polypyrrole (PPy) to result in an electroconductive, acellular patch.

Han et al. (2022) studied the efficacy of cardiac patches of co-cultured rat aortic endothelial cells and cardiomyocytes in fibrin reinforced by MEW scaffolds with stacked fiber walls oriented at 0°, 60°, and 120°. This fiber architecture was inspired by the transition of cellular orientations within the thickness of the myocardium. In an infarcted myocardium *in vivo* rat model, the patches increased the survival to 90% compared to the control group (sham operation), and improved myocardial contractile activity was shown via M-mode echocardiographs.

Montero-Calle et al. (2022) built a computational model to predict the evolution of cardiac patches by correlating cell alignment and functional performance. Their model was informed by cardiac minitissues consisting of iPSC-CM embedded in a hybrid matrix of matrigel reinforced with a box-pore MEW scaffold. In agreement with the model’s predictions, the minitissues displayed advanced maturation and functionality after 28 days, together with a substantial expression of cardiac genes.

## 4 Vascular grafts

Vascular grafts are an important tool of vascular surgery. However, autologous grafts are not always available, and synthetic ones perform poorly in small-diameter settings. Tissue-engineered grafts that build on the MEW platform to provide biomimetic solutions could help tackle this issue.

**FIGURE 2:**
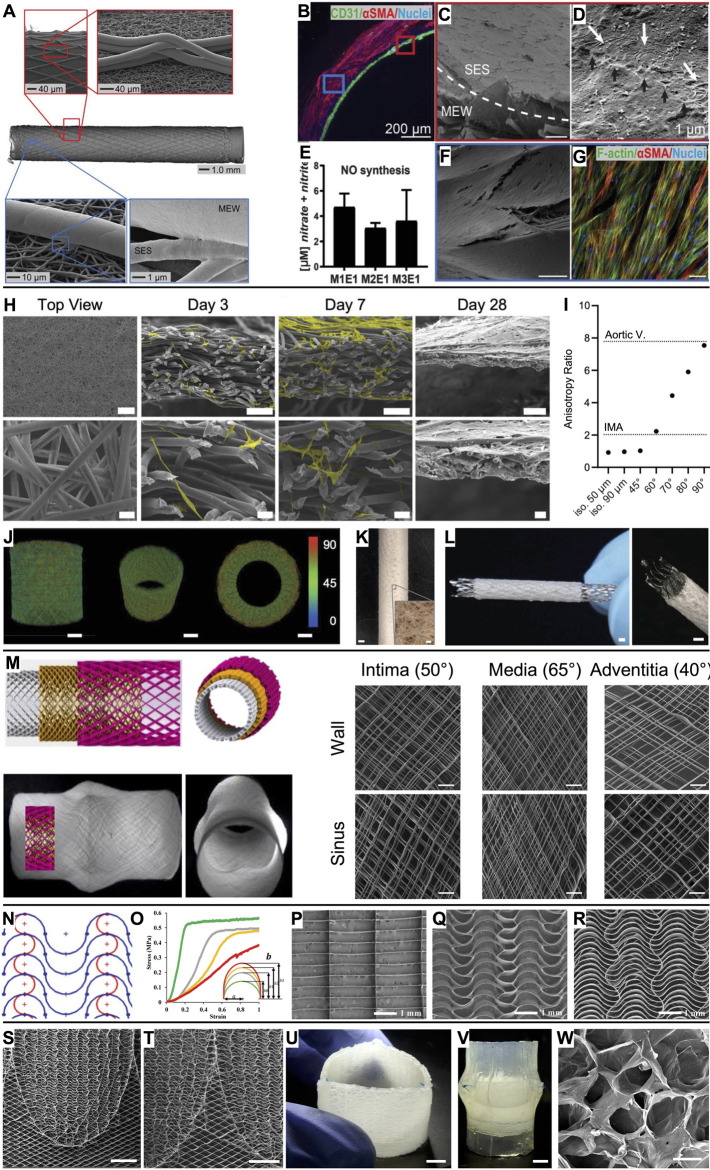
Vascular grafts and heart valves based on MEW scaffolds. **(A)** A bilayered heterotypic scaffold architecture consisting of electrospun nanofibers and aligned MEW fibers. **(B)** While endothelial colony-forming cells (CD31^+^) were cultured on the luminal side of the graft, vSMCs (αSMC^+^) colonized the wall. **(C)** Completely endothelialized electrospun lumen with **(D)** tight cell–cell connections (black arrows). **(E)** Nitric oxide (NO) production from endothelial cells. **(F)** The MEW structures were covered by vSMCs, which **(G)** were oriented along the MEW fibers. **(H)** Microporous scaffold architecture that supports efficient infiltration of smooth muscle cells (scale bars 500 µm, 100 µm, and 20 µm) and **(I)** enables tailored anisotropy. **(J, K)** The design strategy can be translated to seamless tubular scaffolds and **(L)** used to produce covered stents (scale bars 1 mm, inset 100 µm). **(M)** Aortic root model with sinuses of Valsalva and a wall architecture inspired by the native collagen fiber distribution (scale bars 200 µm). **(N, O)** A serpentine fiber architecture with tunable curvature degree enabled tailored mechanical properties. Compared to **(P)** box pore scaffolds, **(Q, R)** serpentine scaffolds outperformed in mimicking the J-shaped stress–strain response of leaflet tissue. **(S, T)** Spatially heterogeneous scaffold with serpentine fiber architecture at the leaflets and diamonds at the interleaflet triangles (scale bars 5 mm). **(U, V)** Composite construct after embedding the scaffold in an ELR hydrogel and suturing it into a silicone aortic root (scale bars 5 mm). **(W)** Salt leaching/gas foaming technique resulted in micropores in the ELR hydrogel for *in situ* TE (scale bar 50 µm). **(A–G)** Scale bars represent 100 µm unless otherwise stated. Adapted with permission from Jungst et al. (2019), CC BY-NC 4.0. **(H–L)** Adapted with permission from Mueller et al. (2023b), CC BY-NC 4.0. **(M)** Adapted with permission from Saidy et al. (2020), CC BY 4.0. **(N–R)** Adapted with permission from Saidy et al. (2019), Wiley. **(S–W)** Adapted with permission from Saidy et al. (2022), CC BY-NC-ND 4.0.

Jungst et al. (2019) presented an integral scaffold design (inner diameter 3 mm) that permits the formation of an intraluminal endothelial cell monolayer surrounded by an outer layer of oriented vascular smooth muscle cells (vSMCs). This was enabled via a bilayered tubular scaffold, comprising an inner layer of randomly oriented electrospun fibers and an outer layer of melt-electrowritten linear microfibers with controlled winding angle (Figure 2A). The heterotypic architecture directed physiological cell organization without the need for soluble factors or bioactivation of the scaffold (Figures 2B–G). Building on this work, Bartolf-Kopp et al. (2024) tuned the ratio of PCL to poly(ester-urethane) during the electrospinning process step of the hybrid constructs to recapitulate the natural J-shaped stress–strain response in native vessels (Young’s modulus of 0.9 ± 0.7 kPa for the cell-seeded hybrid scaffolds, 0.5 ± 0.6 kPa for the internal mammary artery (IMA)). Federici et al. (2023) also investigated the effects of fiber winding angle inspired by the extracellular matrix orientation in the tunica media using a MEW scaffold alone. This biomimetic fiber arrangement promoted neo-tissue formation along the MEW fibers with extracellular matrix deposition preferentially oriented along the pore’s long axis and enabled a biomimetic stress–strain characteristic in the physiological range (up to 10% strain).

Following the *in vivo* tissue engineering approach, Zhi et al. (2022) embedded melt-electrowritten tubular scaffolds subcutaneously in rats to exploit the foreign body response resulting in fibrous encapsulation. These biohybrid constructs performed well in *in vitro* assessments and as abdominal artery replacements in rats. Successful translation to larger animal models (canines and sheep) underlines this as a promising approach for a future alternative to autologous vessel replacements. While in these studies (Bartolf-Kopp et al., 2024; Federici et al., 2023; Jungst et al., 2019; Pickering et al., 2022; Zhi et al., 2022) the MEW fiber architecture was a simple diamond 172 pattern, others reported the design of tubular auxetic (Paxton et al., 2020a) and nonlinear (McCosker et al., 2022) designs that could potentially allow for combined longitudinal and radial growth. Also, serpentine fiber patterns have been demonstrated to enable tubular scaffolds with compliance of 12.9 ± 0.6% (100 mmHg)^−1^ (Weekes et al., 2023), which is the physiological range of the IMA, 11.5 ± 3.9% (100 mmHg)^−1^ (Konig et al., 2009). Performing the fiber deposition process on patient-specific water-soluble polyvinyl alcohol (PVA) molds will lead to further anatomically relevant tubular constructs (Brooks-Richards et al., 2022).

MEW scaffolds have been mainly macroporous due to entrapped charge carriers in the fibers that prevent accurate fiber placement below a fiber diameter-dependent interfiber distance (Ding et al., 2019; Kim et al., 2021; Tourlomousis et al., 2017). Therefore, MEW scaffolds have been used as mechanical reinforcement that must be combined with a secondary biomaterial that provides the microporosity required for cellular infiltration following the *in situ* TE paradigm. To overcome this, Mueller et al. (2023b) developed a design strategy that results directly in microporous MEW scaffolds and allows tailoring the directional anisotropy to a wide range of cardiovascular tissues such as the IMA and the aortic valve leaflets (Figures 2H–K). Furthermore, this approach decouples fiber diameter from pore size (in contrast to electrospinning, where they correlate) and can be applied to both flat and tubular scaffold architectures. Also, covered stents can be fabricated with this approach (Figure 2L). First attempts towards purely melt-electrowritten stents were fabricated from PCL mechanically reinforced with reduced graphene oxide to increase their flexural stiffness (Somszor et al., 2020). How the mechanical properties would evolve with progressing PCL degradation remains to be investigated.

Moving to larger vessel diameters, Saidy et al. (2020) presented a melt-electrowritten aortic root featuring sinuses of Valsalva with a triphasic fiber architecture inspired by the collagen fiber distribution in the intima (50°), media (65°), and adventitia (40°) (Figure 2M). As the aortic roots were also fabricated onto a 3D-printed target obtained from the patient’s data, the scaffolds could potentially be used in personalized external aortic root support (PEARS) procedures.

## 5 Heart valves

Heart valves are characterized by mechanical anisotropy, a nonlinear stress–strain relationship, viscoelasticity, and spatial heterogeneity, and, therefore, they particularly benefit from the capabilities of MEW for complex scaffold designs.

Inspired by the wavy collagen fibers in the extracellular matrix, Saidy et al. (2019) fabricated scaffolds with a serpentine fiber architecture to recapitulate the J-shaped stress–strain response of native leaflet tissue (e.g., high tensile modulus 1 MPa (radial) and 5 MPa (circumferential) for the MEW scaffolds and 2.3 MPa (radial) and 9.9 MPa (circumferential) for native aortic valve tissue (Pham et al., 2017)) (Figures 2N–R). Tuning the curvature degree, interfiber spacing, and layer number enabled tailored mechanical characteristics, anisotropy, out-of-plane flexibility, and viscoelastic behavior closely matching human tissue. The scaffolds’ suitability for heart valve engineering was tested by embedding them in fibrin and suturing them as single leaflets to form a trileaflet valve in a silicone aortic root model. The valve complied with ISO 5840 standards under aortic conditions (International Organization for Standardization, 2021). Similarly, Mirani et al. (2023) exploited computational modeling and design of experiments to produce sinusoidal fiber patterns with prescribed biaxial mechanics mimicking the tissue characteristics of an adult aortic valve, a pediatric pulmonary valve, and the pediatric pericardium. Embedding the MEW scaffolds in a cell-laden fibrin hydrogel resulted in tissue sheets that were sutured into trileaflet valves that performed well under pulmonary pressure conditions in a pulse duplicator bioreactor (Mirani et al., 2023).

With the motivation of fabricating a tubular trileaflet valve, Saidy et al. (2022) presented a spatially heterogeneous tubular scaffold design that featured the established serpentine architecture in the leaflet regions, while the interleaflet triangles and annulus region showed a diamond architecture (±25° linear fibers) to allow for diameter changes according to the dynamic circulatory pressure conditions (Figures 2S,T). Subsequently, the macroporous scaffolds were embedded in an elastin-like recombinamer (ELR) hydrogel containing a porogen so that a microporous composite for *in situ* TE was obtained by salt leaching/gas foaming technique (Figures 2U–W). These valves were sutured in a silicone aortic root using the single point attachment commissure (SPAC) technique and were in accordance with ISO 5840 requirements when tested in a flow-loop system under aortic conditions.

Vernon et al. (2022) focused on interfaces in melt-electrowritten heart valve scaffolds, specifically on the interleaflet triangle to leaflet region. Compared to an overlapping or suture-like regional link, the authors advocate for continuous fiber transitions and gradient porosities, as this will result in superior tensile and flexural properties while better mimicking the collagen orientation, density, and recruitment in native valves.

This body of work impressively demonstrates the potential of engineering heart valves based on MEW scaffolds.

## 6 Technological and material advancements

Mimicking native tissues often requires scaffold design using multiple scales and materials (Castilho et al., 2020). Therefore, converging biofabrication techniques have led to the hybridization of MEW with multiple technologies. Examples of hybrid MEW + X approaches include those where MEW has been combined with molding (Saidy et al., 2022; 2019) and extrusion-based bioprinting of hydrogels (Ainsworth et al., 2023; de Ruijter et al., 2019) to fill the macropores of MEW scaffolds and to introduce cells as living components. In other cases, MEW has been hybridized with volumetric bioprinting (Größbacher et al., 2023), solution electrospinning (Bartolf-Kopp et al., 2024; Jungst et al., 2019), and melt electrospinning (Großhaus et al., 2020). Recently, translating the electric-field driven fiber formation to the widely established filament-based additive manufacturing technology (fused filament fabrication, FFF) enabled the fabrication of multi-scale scaffolds by combining macroscale FFF prints with microfibrous MEW scaffolds manufactured by a single print head (Mueller et al., 2023a). This approach also had the motivation of making MEW more accessible by a straightforward modification of commercially available FFF printers.

Given the technological progress and increasing number of *in vivo* studies with MEW scaffolds, clinical translation of MEW products is within reach, yet it will be accompanied by the need for high throughput fabrication with excellent quality. In this context, MEW setups with multiple print heads working in parallel (Wunner et al., 2019), *in situ* process monitoring (Collier et al., 2022; Mieszczanek et al., 2021a) and, consequently, the ability to self-correct process parameters based on machine learning (Mieszczanek et al., 2021b) will be of great importance.

MEW is an AM technology and, as such, holds the potential to produce highly complex, patient-specific scaffold geometries. However, MEW is inherently bound to deposit the fibers onto a collector, which limits design freedom and poses significant challenges when depositing fibers on curving surfaces that stray from a single plane of deposition (i.e., “out-of-plane” fiber deposition). Recent attempts to perform MEW onto out-of-plane geometries point to the difficulty of controlling process parameters such as the electric field and the print speed on complex collector geometries (Brooks-Richards et al., 2022; Luposchainsky et al., 2022; Mueller et al., 2023a; O’Connell et al., 2021; Peiffer et al., 2020; Saha et al., 2021; Saidy et al., 2020). Solving these challenges will require specific advancements in both hardware and software components, including versatile toolpath generators (Paxton et al., 2020b).

In parallel, the biomaterial library accessible to MEW is also quickly expanding. In addition to PCL as the undoubted gold standard material, researchers are introducing new polymers, composites, and bioactive coatings for MEW scaffolds. This progress has been extensively reviewed elsewhere (Kade and Dalton, 2021; Saiz et al., 2024). Incorporating ultrasmall superparamagnetic iron oxide nanoparticles as contrast agents into the PCL microfibers added the option for non-invasive magnetic resonance imaging of MEW scaffolds (Mueller et al., 2021). This approach was further exploited by using metal-organic frameworks (MOFs) as additives that can provide multiple combined functions (Mansi et al., 2023). Like this, MEW scaffolds that were simultaneously MRI visible, antimicrobial, and drug-loaded were obtained with only one additive. Alternative approaches for introducing imaging capabilities to MEW scaffolds exploited PCL labeled with fluorophores or near-infrared region II dyes (Hall et al., 2024; Jing et al., 2021).

Of particular interest for cardiovascular applications is the use of the electroactive polymer poly(vinylidene fluoride) (PVDF) with incorporated carbonyl iron particles, which offers the potential for magnetoactive cell stimulation (Kade et al., 2022). Resistance against bacterial infection and biofilm formation is particularly important for successful clinical translation and has been tackled by loading antibiotics (Bai et al., 2020; Mathew et al., 2023) or silver nanoparticles (Du et al., 2022) into the polymers or by coating calcium phosphate nanoparticles onto melt-electrowritten fibers (Abdal-hay et al., 2023). Ainsworth et al. (2022) coated PCL scaffolds covalently with TGF-β1 after (reagent-free) plasma treatment to enhance hydrophilicity and enable cytokine loading for improved tissue regeneration, while thiol and carbodiimide chemistry was also used to conjugate peptides to MEW PCL scaffolds (Mirzaei et al., 2023). Ryma et al. (2021) combined flow-directed polymer phase separation during MEW with the selective dissolution of the matrix polymer to obtain nanofiber bundles with structural similarity to native collagen I from PCL/poly(vinylacetate) blends. These fibrillar structures were capable of highly efficient topographic immunomodulation. Furthermore, the potential of drug-loaded MEW scaffolds was demonstrated with water-soluble indomethacin loaded in a poly(2-oxazoline)-based triblock copolymer for sublingual drug delivery (Keßler et al., 2022).

## 7 Conclusion

Given MEW’s capability for precise control over fiber diameter and deposition, we see great potential to further drive TE research towards functional regenerative implants by employing complex fiber-based scaffold architectures with tailored properties and also opportunities for future *in vitro* models. Vascular constructs have been realized from the scale of capillary networks by exploiting sacrificial MEW fibers embedded in various matrices to small-diameter vascular grafts via tubular MEW constructs. Both cardiac patches and heart valve scaffolds have been designed to exploit application-specific structure-function relationships of MEW scaffolds.

Although a plethora of MEW scaffold microarchitectures have been investigated, MEW has yet to demonstrate its full potential toward complex scaffold macrogeometries, such as bifurcating vessels and multicurvature heart valves. Reaching these milestones will open new avenues for the development of a broad range of tissues beyond the cardiovascular field. MEW is a powerful technique that provides access to a previously unavailable design space. Intriguing new opportunities are presented in this review, including hybrid biofabrication approaches (i.e., MEW + X) that synergistically provide novel solutions to achieve the ultimate goal: improved therapies that benefit patients.
